# Cryptogenic organizing pneumonia—Results of treatment with clarithromycin versus corticosteroids—Observational study

**DOI:** 10.1371/journal.pone.0184739

**Published:** 2017-09-25

**Authors:** Elżbieta Radzikowska, Elżbieta Wiatr, Renata Langfort, Iwona Bestry, Agnieszka Skoczylas, Ewa Szczepulska-Wójcik, Dariusz Gawryluk, Piotr Rudziński, Joanna Chorostowska-Wynimko, Kazimierz Roszkowski-Śliż

**Affiliations:** 1 III Department of Lung Disease National Tuberculosis and Lung Diseases Research Institute, Warsaw, Poland; 2 Pathology Department National Tuberculosis and Lung Diseases Research Institute, Warsaw, Poland; 3 Radiology Department National Tuberculosis and Lung Diseases Research Institute, Warsaw, Poland; 4 Geriatrics Department National Institute of Geriatrics, Rheumatology and Rehabilitation, Warsaw, Poland; 5 Thoracic Surgery Department National Tuberculosis and Lung Diseases Research Institute, Warsaw, Poland; 6 Laboratory of Molecular Diagnostics and Immunology National Tuberculosis and Lung Diseases Research Institute, Warsaw, Poland; Medizinische Hochschule Hannover, GERMANY

## Abstract

**Background:**

Cryptogenic organizing pneumonia (COP) is a clinicopathological syndrome of unknown origin. Corticosteroids are the standard treatment, but clarithromycin (CAM) is also effective. The aim of this observational retrospective study was to compare the results of CAM versus prednisone (PRE) treatment in patients with biopsy-proven OP without respiratory insufficiency.

**Material and methods:**

In a 15-year period, 40 patients were treated with CAM (500 mg twice daily orally for 3 months) and 22 with PRE (mean initial dose of 0.67 ± 0.24 mg/kg/d for a mean of 8.59 ± 3.05 months).

**Results:**

The clinical presentation, laboratory, and radiological findings did not differ markedly between patients treated with CAM and PRE, with the exception of a higher frequency of sweats (55% vs. 23%; *p* < 0.015), ground glass opacities (95% vs. 50%; *p* <0.0001) and nodular lesions (45% vs. 18%; *p* = 0.036) in the CAM group. A complete response was achieved in 35(88%) patients treated with CAM and in all treated with PRE. Patients treated with PRE relapsed more frequently than those treated with CAM (54.5% vs. 10%; *p* < 0.0001). Corticosteroid-related adverse events were noticed in 8(6.5%) patients (with one death), but CAM caused only one (2.5%) allergic reaction. A FVC >80% identified patients who might be successfully treated with CAM with a sensitivity of 60% and a specificity of 88.57% (AUC 0.869; 95% CI 0.684–1; *p* = 0.008); the figures for the FEV1 were >70%, a sensitivity of 60%, and a specificity of 91.43% (AUC 0.809; 95%CI 0.609–1; *p* = 0.027).

**Conclusions:**

CAM can be used to treat COP patients in whom the pulmonary function parameters are within normal limits. Such therapy is shorter, better tolerated, and associated with fewer adverse events and relapses than is PRE. However, the therapy is ineffective in some patients.

## Introduction

Organizing pneumonia (OP) is a distinct clinicopathological entity that results from the pulmonary reaction to various injuries. It is classified as cryptogenic OP (COP) when the triggering factor is unknown or secondary. Causes of the secondary form are mainly infections, exposure to toxic substances, drugs, connective tissue diseases, malignancies, autoimmune diseases, bone marrow or organ transplantation, and radiotherapy [1–18]. Clinical features include subacute flu-like disease onset with cough, slight fever, weakness, progressive exertional dyspnea, sweats, weight loss, and migratory opacities (often bilateral) on chest X-rays [1–18]. Histologically, OP is defined by an organized inflammatory exudate in alveoli and small bronchioli in the shape of characteristic polyps and variable degrees of interstitial inflammation; the lung parenchyma is preserved [2,8]. The prognosis of the disease is usually good, and spontaneous regression of COP occurs in about 5% to 10% of cases [2,11–13]. Corticosteroids are the standard treatment for OP [1–20]. Regression is observed quickly after treatment initiation; however, the tapering of steroids can induce relapse in about 50% of cases [12,14,20]. Other immunosuppressive therapies, such as cyclophosphamide, cyclosporine, and macrolides, also seem to be effective [2,18–26].

Macrolides have specific activity against many Gram-positive and Gram-negative bacteria; they also demonstrate nonspecific anti-inflammatory effects [27–39]. Concentrations of inflammatory mediators, such as tumor necrosis factor (TNF), interleukin 8 (IL-8), and interleukin 1b (IL-1b), produced by the endothelium and respiratory tract cells are diminished during treatment with these antibiotics [33–39]. These cytokines are essential chemotactic factors for neutrophils and play important roles in the pathogenesis of COP[2,36,39–41]. Previous data showed potential activities of macrolides in patients with COP [21–26,40,41]. The aim of this study was to compare the results of CAM versus prednisone (PRE) in patients with OP.

## Material and methods

From 1999 to 2014, 76 patients with OP were diagnosed in our Department. However, we here (retrospectively) analyze only 62 patients who were respiratory sufficient. The observation period ended in June 2016. A diagnosis of COP was established in 57 patients; in 5 patients, OP was secondary to breast cancer radiotherapy. We earlier presented data on 43 patients[13,25,40,41].

All patients were initially treated with prednisone beginning at 1 mg/kg/d with subsequent dose reduction, for 12 (rarely 18) months. After the report of Lazor et al. appeared, we reduced the starting dose to 0.5 mg/kg/d and the course to (usually) 6 months [12]. All patients given CAM were informed that steroids were the standard treatment for OP and that the effectiveness of CAM was not sufficiently proven. All volunteered to take CAM in preference to PRE and gave written informed consent. Patient data were not anonymized. All patients were evaluated regarding their medical history, smoking, additional diseases, consumption of medicines, symptoms, and symptom duration. Routine blood and urine tests, microbiological sputum and/or bronchial washing examinations, immunological and cytological assessments of bronchoalveolar lavage (BAL) specimens, ultrasound examinations of the abdomen and thyroid gland, and pulmonary function tests were conducted. The presence of connective tissue diseases, antinuclear antibodies, antithyroid antibodies, antineutrophil cytoplasmic antibodies, rheumatoid factor, and serological tests for Mycoplasma pneumoniae, Chlamydia pneumoniae, and Legionella pneumophila were assessed. Additional tests for adenovirus, cytomegalovirus, influenza virus, and parainfluenza virus, respiratory syncytial virus, and hepatitis B and C virus infection were performed in selected cases. Chest X-ray and high-resolution computed tomography (CT) scans were available in all cases and were reviewed by an experienced radiologist (I.B.). Two pulmonary pathologists analyzed all lung specimens independently (R.L. and E.Sz.). The following criteria were required for COP diagnosis: pulmonary infiltrations suggestive of OP in radiological examinations, characteristic changes in lung biopsy specimens, negative microbiological and cytological analysis of BAL fluid and/or sputum, and exclusion of other possible causes of OP. CAM was orally administered at a dose of 500 mg twice daily for 3 months and PRE at an initial dose of 1.0 to 0.3 mg/kg/d. Patients treated with CAM were assessed every month during treatment and reevaluated at the end of treatment. Subsequently, during the first year, patients were examined after 3 and 6 months and then once per year for 3 to 5 years. Patients treated with PRE were usually evaluated after 1, 3, 6, and 12 months of treatment and subsequently once per year. Relapsed patients underwent continued observation. Regression of COP was defined as resolution of symptoms, decreases in serum inflammatory marker concentrations, and complete or nearly complete resolution of pulmonary infiltrates on CT scans. Relapses were defined as the presence of symptoms and new pulmonary opacities on CT chest imaging suggestive of OP, with clinical and laboratory exclusion of other possible causes of the lesions. Patients in whom CAM was ineffective were given oral PRE at 0.5 mg/kg for 6 weeks, followed by tapering over a period of 6 months.

This study was supported by the National Tuberculosis and Lung Diseases Research Institute grant no. 7.3, and approved by the National Tuberculosis and Lung Diseases Research Institute Bioethics Committee.

### Statistical methods

Statistical analysis was performed using R software. The Shapiro-Wilk test was employed to assess the normality of distribution and the F-test to explore the homogeneity of variances between the two groups. Continuous variables were compared using the unpaired Student’s t-test, or the Mann–Whitney U-test, when the variances were equal or unequal respectively. Pearson χ^2^ test (with or without the Yates modification), the V-squared test, and Fisher’s exact test were used (as appropriate) to create contingency tables. Multivariate logistic regression was performed to explore the influence of variables on treatment failure and/or relapse.. Selected parameters were subjected to ROC analysis. A *p*-value <0.05 was considered reflect statistical significance. All p values are two-sided and unadjusted for multiple testing.

## Results

### Clinical, radiological, laboratory, and pulmonary function results

#### All patients

The studied population comprised 46 (74%) women and 16 (26%) men. The mean age at diagnosis was 55.92 ± 10.15 years (range, 31.0–74.0 years) (Table 1).

**Table 1:** Characteristics of patients with organizing pneumonia.

	All No. (%)	Patients treated with clarithromycin–No. (%)	Patients treated with prednisone–No. (%)	*p*- value
**Gender–All**	62	40 (65)	22 (35)	0.426
Female	46 (74)	31 (77.5)	15 (68)	
Male	16 (26)	9 (22.5)	7 (32)	
Mean age (years) ± SD	55.92 ± 10.15	57.05 ± 9.84	53.86 ± 10.59	0.239
Age range (years)	31–74	36–72	31–74	
**Smoking status**				0.095
Nonsmokers	44 (71)	31 (77.5)	13 (59)	
Ex-smokers	9 (14.5)	4 (10)	5 (23)	
Smokers	9 (14.5)	5 (12.5)	4 (18)	
**Concomitant diseases**				
Goiter	22 (35)	17 (43)	5 (23)	0.122
Hypertension	22 (35)	17 (43)	5 (23)	0.122
COPD	3 (5)	2 (5)	1 (4.5)	1
Diabetes mellitus	4 (6)	1 (2.5)	3(14)	0.243
Ischemic heart disease	4 (6)	3 (7.5)	1 (4.5)	1
Stomach/duodenal ulcers, GERD	6 (10)	5 (12.5)	1 (4.5)	1
Depression	2 (3)	2 (5)	0	0.752
Asthma	3 (5)	2 (5)	1 (4.5)	1
Breast cancer	5 (8)	3 (7.5)	2 (9)	1
**Biopsy**				
Open lung biopsy	37 (60)	16 (40)	21 (95.5)	**0.00002**
Transbronchial lung biopsy	25 (40)	24 (60)	1 (4.5)	

COPD chronic obstructive pulmonary disease; GERD gastro-esophageal reflux disease

There were 44 (71%) nonsmokers, 9 (14.5%) smokers, and 9 (14.5%) ex-smokers. Histological diagnoses were based on assessment of 37(60%) specimens obtained by open lung biopsy, and 25(40%) specimens from transbronchial biopsy. The most frequently diagnosed concomitant diseases were arterial hypertension (35%), goiter (35%), diabetes mellitus (6%), ischemic heart disease (6%), chronic obstructive pulmonary disease (5%), and GERD with or without a duodenal ulcer (10%) (Table 1). The most common symptoms were weakness (90%), fever (76%), cough (79%), dyspnea (55%), weight loss (50%), sweats (43.5%), chest pain (29%), and sputum expectoration (27%) (Table 2). The mean duration of symptoms before diagnosis was 3.31 ± 2.57 months (range, 1.0–9.0 months).

**Table 2:** Clinical and radiological presentations of patients with organizing pneumonia.

	All		Patients treated with clarithromycin		Patients treated with prednisone		*p*- value
	No. of examined patients	No (%)	No. of examined patients	No (%)	No. of examined patients	No (%)	
**Symptoms**							
Fever	62	47 (76)	40	29 (72.5)	22	18 (82)	0.416
Cough	62	49 (79)	40	32 (80)	22	17 (77)	1
Sputum	62	17 (27)	40	12 (30)	22	5 (23)	0.542
Dyspnea	62	34 (55)	40	22 (55)	22	12 (54.5)	0.973
Weakness	62	56 (90)	40	38 (95)	22	18 (82)	0.218
Weight loss	62	31 (50)	40	22 (55)	22	9 (41)	0.288
Sweat	62	27 (43.5)	40	22 (55)	22	5 (23)	**0.015**
Chest pain	62	18 (29)	40	11 (28)	22	7 (32)	0.722
**Duration of symptoms**							
Range (months)	62	1–9	40	1–9	22	1–9	0.624
Mean ± SD (months)	62	3.31 ± 2.57	40	3.15 ± 2.07	22	3.59 ± 2.4	
**RTX. HRCT**							
Bilateral infiltrations	62	61 (98)	40	39 (98)	22	22 (100)	0.759
Unilateral infiltrations	62	1 (2)	40	1 (2.5)	22	0	1
Air bronchogram	62	61 (98)	40	40 (100)	22	21 (95.5)	0.759
Ground glass opacities	62	49 (79)	40	38 (95)	22	11 (50)	**0.0001**
Migration	62	40 (64.5)	40	29 (72.5)	22	11 (50)	0.079
Nodules	62	22 (37.5)	40	18 (45)	22	4 (18)	**0.036**
Reticular lesions	62	10 (16)	40	7 (17.5)	22	3 (14)	1
Pleural fluid	62	2 (3)	40	1 (2.5)	22	1 (4.5)	0.188
Lymph node enlargement	62	8 (13)	40	3 (7.5)	22	5 (23)	0.972
**Mean ESR (mm/h)**	55	74.4 ± 35	35	71.6 ±35.8	20	74.1 ± 38.2	0.806
**Mean leukocyte count (/mL)**	62	7976 ± 2099	40	8036 ± 2406	22	7867 ± 2422	0793
**Mean eosinophil count (/mL)**	59	240 ± 0.27	39	239 ± 0.3	20	220 ± 0.16	0.591
**ANA >1:160**	58	8 (14)	40	6 (15)	18	2 (11)	1
**RF-positive**	60	3 (5)	40	1 (2.5)	20	2 (10)	0.59
**Antithyroid peroxidase antibody-positive**	47	6 (13)	31	4 (13)	16	2 (13)	1
**Antithyroglobulin antibody-positive**	47	3 (6.5)	31	2 (6)	16	1 (6)	1

ANA Antinuclear antibody; ESR Erythrocyte sedimentation rate; HRCT High-resolution computed tomography; RF Rheumatoid factor; RTX Chest X-ray examination

Sixty-one (98%) patients had radiological changes consistent with the typical pattern of interstitial attenuation with air bronchogram. Ground glass opacities were present in 49 (79%) patients. Nodular lesions (35.5%), diffuse reticular changes (16%), lymph node enlargement (13%), and pleural fluid (3%) were less frequent radiological findings. A migratory pattern of lesions was observed in 64.5% of patients with OP (Table 2). All patients had elevated erythrocyte sedimentation rates (mean, 74.4 ± 35 mm/h). Nonspecific elevations of antinuclear antibody titers and rheumatoid factor were noted in 8(14%) of 58 patients and 3(5%) of 60 patients, respectively. Increased serum concentrations of antithyroid peroxidase and antithyroglobulin antibodies were found in 6 (13%) and 3(6.5%) of 47 patients, respectively (Table 2). Spirometry data were available in all patients; body plethysmography and determination of the diffusing capacity of the lungs for carbon monoxide were performed in 59 (95%) and 53 (85.5%) patients respectively (Table 3). Fifteen (24%) patients showed a vital capacity of <80% pred., and 11 (18%) patients had a forced expiratory volume in 1 s of <70% pred. A decreased TLC <80% predicted was seen in 7(12%) patients, and 6 (10%) patients showed the RV <80% predicted. Hyperinflation defined as a TLC or an RV >120% the predicted values, was evident in 7 and 12% of patients, respectively. A decreased diffusing capacity of the lungs for carbon monoxide was the most frequently noted disturbance (45% of all patients). No patient displayed significant resting hypoxemia (PaO2 <60 mmHg) or hypercapnia (PaCO2 >45mmHg).

**Table 3:** Pulmonary function test data from patients with organizing pneumonia.

	All		Patients treated with clarithromycin		Patients treated with prednisone		P—value
	No. of examined patients	No. (%) 62	No. of examined patients	No. (%) 40	No. of examined patients	No. (%) 22	
FVC mean % pred. ± SD	62	92.9 ± 20.83	40	93.98 ± 19.23	22	91 ± 23.8	0.357
FVC <80% pred.	62	13 (21)	40	7 (17.5)	22	8 (36.5)	0.097
FEV1 mean % pred. ± SD	62	88.32 ± 14.14	40	89.48 ± 15.3	22	86.22 ± 20.95	0.486
FEV1 <70% pred.	62	11 (18)	40	6 (15)	22	5 (23)	0.446
FEV1% mean % pred. ± SD	62	92.08 ± 16.97	40	90.92 ± 13.12	22	94.18 ± 11.66	0.196
FEV1% <80% pred.	62	11 (18)	40	8 (20)	22	3 (14)	0.502
TLC mean % pred. ± SD	59	97.08 ± 20.8	37	96.78 ± 17	22	97.6 ± 14.78	0.854
TLC <80% pred.	59	7 (12)	37	5 (13.5)	22	2 (9)	NS
TLC >120% pred.	59	4 (7)	37	2 (5.4)	22	2 (9)	NS
RV mean %pred. ± SD	59	100.96 ± 15.6	37	100.9 ± 20.39	22	101 ± 12.2	0.989
RV <80% pred.	59	6 (10)	37	5 (13.5)	22	1 (4.5)	NS
RV >120% pred.	59	7 (12)	37	6 (16)	22	1 (4.5)	0.24
DLCO mean % pred. ± SD	53	73.07 ± 22.63	34	72.2 ± 14.97	19	74.63 ±19.53	0.615
DLCO <70% pred.	53	24 (45)	34	16 (47)	19	8 (42)	0.728
PaO2 mean (mmHg) ± SD	62	73.61 ± 4.24	40	73.4 ± 7.76	22	74 ± 10.56	0.757
PaCO2 mean (mmHg) ± SD	62	36.27 ± 2.12	44	36.45 ± 3.14	32	35.95 ± 3.62	0.968

FVC Forced Vital Capacity; FEV1 Forced Expiratory Volume in 1 s; TLC Total Lung Capacity; RV Residual Volume; DLCO Diffusion Lung Capacity for Carbon Monoxide; PaO2 Partial pressure of oxygen in arterialized blood; PaCO2 Partial pressure of carbon dioxide in arterialized blood; pred. predicted; SD standard deviation

#### Comparison between patients treated with CAM and PRE

There was no difference in sex, age, smoking, or concomitant diseases between the CAM- and PRE treated patients (Table 1). Clinical symptoms were equally distributed between the two groups with the exception of sweats, which were observed more frequently in patients treated with CAM than PRE (55% vs. 23%; *p* = 0.015) (Table 2). The duration of symptoms was similar in patients treated with PRE and CAM (3.59 ± 2.4 vs. 3.15 ± 2.07 months; *p* = 0.623). Ground glass opacities (95% vs. 50%; *p* = 0.0001) and nodular lesions (45% vs. 18%; *p* = 0.036) were more frequently seen in patients treated with CAM than PRE (Table 2). There was no difference in laboratory data or mean values of pulmonary function tests between groups (Table 3).

### Results of treatment, adverse events, and observations

A complete response was achieved in 35 (88%) patients treated with CAM and in all treated with PRE. PRE was orally administered at a mean initial dose of 0.67 ± 0.24 mg/kg for a mean period of 8.59 ± 3.05 months. CAM treatment was ineffective in five patients (12%), and PRE at an initial oral dose of 1 mg/kg was therefore initiated. Sixteen (26%) patients experienced relapse, 5 (8%) of them multiple times. Patients treated with PRE relapsed significantly more frequently than patients treated with CAM (54.5% vs. 10%; p < 0.0001), and multiple relapses were also more frequent (18% vs. 2.5%; p = 0.049) (Table 4). Relapses were treated successfully with CAM in 1 patient from the CAM group (3 relapses) and in 9 patients (21 relapses) from the PRE group. Prednisone was administered to treat relapses in 3 patients from the CAM group and in 9 patients (13 relapses) who received it as the initial treatment. Concomitant treatment with CAM and PRE was administered to patients who experienced multiple relapses: four patients from the PRE group and one from the CAM group.

**Table 4:** Results of treatment and adverse events in patients with organizing pneumonia.

	All No. (%)	Patients treated with clarithromycin No. (%)	Patients treated with prednisone No. (%)	*p*- value
	**62**	**40**	**22**	
**Relapse**	16 (26)	4 (10)	12 (54.5)	**0.0001**
**Multiple relapses**	5 (8)	1 (3)	4 (18)	0.049
**Treatment failure**	5 (8)	5 (12)	0	0.214
**Death as an adverse event of treatment**	**1 (2)**	0	**1 (4.5)**	1
**Adverse events**	9 (15)	1(2.5)	8 (32)	**0.00006**
Hypertension	5 (8)	0	4 (18)	0.02
Diabetes	2 (3)	0	2 (9)	0.235
Increase in body weight >10%	5 (8)	0	5 (23)	0.007
Bone fracture	1 (2)	0	1 (4.5)	0.759
Upper respiratory tract infection	8 (13)	0	8 (32)	**0.0002**
Urinary tract infection	2 (3)	0	2 (9)	0.235
Allergic reaction	1 (2)	1 (2.5)	0	0.759
Gastrointestinal bleeding	1 (2)	0	1 (4.5)	0.759
Pulmonary embolism	1 (2)	0	1 (4.5)	0.759
**Observation period**				
**Range (months)**	12–192	12–114	19–192	
**Mean** ± **SD**	60.26 ± 33.53	56.55 ± 24.43	67.0 ± 45.6	0.994
**Diseases diagnosed during observation**				
Sarcoidosis	1(2)	1 (2.5)		0.759
Thyroid cancer	1(2)		1 (4.5)	0.759

Of the five patients with OP after breast cancer radiotherapy three were treated successfully with CAM without relapses but two who were treated with PRE relapsed. One of these patients was treated with CAM with remission but the second relapsed repeatedly. Corticosteroid-related adverse events were noted in 8 (36.5%) patients. Patients with arterial hypertension and diabetes mellitus required escalation of antihypertensive and antidiabetic therapies. Two patients required insulin therapy. An increase in body weight (>10% of previous weight) was noted in 5 (23%) patients; 8 (32%) had upper respiratory tract infections and 2 (9%) had urinary tract infections during PRE treatment. One patient treated with PRE experienced vertebral compression and one female died from fulminant gastrointestinal bleeding (Table 4). Of all patients treated with CAM only one developed an allergic reaction.

Single-factor logistic regression analysis identified the FVC (OR 0.905; 95% CI 0.84–0.974; *p* = 0.008) and the FEV1 (OR 0.92; 95% CI 0.854–0.991. *p* = 0.029) as factors significantly influencing the response to CAM (Table 5). Multivariate logistic regression analysis was performed to find the optimal model predicting this response. The only factors listed in Table 5 that were significant were the FVC% pred. and FEV1% pred. In a model using only these factors, only the FVC% pred. was essential (OR 0.912; 95% CI 0.84–0.991; *p* = 0.03) (Table 6).

**Table 5:** Response to CAM; single-factor logistic regression analysis.

Variable	OR	-95%CI	+95%CI	*p*- value
Age	1.040	0.940	1.151	0.444
**FVC % pred**.	**0.905**	**0.840**	**0.974**	**0.008**
**FEV1% pred**.	**0.920**	**0.854**	**0.991**	**0.029**
TLC% pred.	0.945	0.886	1.008	0.088
DLCO %pred.	0.972	0.907	1.041	0.417
PaO_2_ (mmHg)	0.916	0.795	1.055	0.223
TBLB	0.130	0.013	1.3	0.083
Time to diagnosis	0.898	0.534	1.510	0.684
Observation time	1.001	0.963	1.041	0.949

Pulmonary function tests data obtained at diagnosis:

FVC Forced Vital Capacity;

FEV1 Forced Expiratory Volume in 1s;

TLC Total Lung Capacity;

DLCO Diffusion Lung Capacity for Carbon Monoxide;

PaO2 Partial pressure of oxygen in arterialized blood;

pred. predicted;

TBLB Transbronchial lung biopsy;

OR Odds Ratio;

CI Confidence Interval

**Table 6:** Response to CAM: Multivariate logistic regression.

Variables analyzed	*p-* value for model	Significant parameter	*p*-value for parameter	OR	-95CI	+95CI
FVC % pred. FEV1% pred.	0.00126	FVC % pred.	0.03	0.912	0.84	0.991

FVC Forced Vital Capacity; FEV1 Forced Expiratory Volume in 1s; pred. predicted; OR Odds Ratio; CI Confidence Interval

In addition, the univariate models were subjected to ROC analysis. We used two methods to select optimal cut-off points: the “best Youden value” and the “point nearest to the top left” methods. In addition, we assigned arbitrary cut-offs of 80% and 70% to the FVC% pred. and FEV1% pred., respectively. We calculated positive and negative likelihood ratios for each parameter. Both calculated AUCs differed significantly from 0.5, indicating that both parameters were good predictors of the response to CAM treatment. However, the AUC confidence interval for FVC% pred. was narrower and lower than that for FEV1% pred. All PPVs were low, indicating that many positives (thus, predicted to fail treatment) were false using these tests. A high NPV indicates that a negative result is a true-negative and CAM treatment should be effective. Good cut-off points have high LR(+) and low LR(-) values (in practice >5 and <0.25, respectively). Our aim when evaluating respiratory parameters was to identify good candidate predictors of the CAM treatment response. Minimizing the probability of failure, then, took center stage; the cut-off point should have a minimal LR(-). After considering these issues, we determined that the best FVC% pred. cut-off point was 80%, with a sensitivity of 60% and a specificity of 89%. The FEV1% pred. was not as good a parameter, consistent with the results of logistic regression, being associated with a cut-off of 70%, a specificity of 96%, and a sensitivity of 60% (Table 7). In summary, the best candidates for CAM treatment were those with normal lung function parameters. Higher PFT values translated into a higher probability of a good outcome.

**Table 7:** ROC analysis of parameters using the selected cut-off points.

	AUC (95%CI)	*p*- value*	Cut-off	Method**	Sensitivity	Specificity	PPV	NPV	LR(+)	LR(-)
FVC% pred.	0.869 (0.684–1)	0.008	87.5	1	80.00%	77.14%	11.55%	96.43%	3.50	0.26
			61.5	2	60.00%	100.00%	8.92%	94.59%	∞	0.40
			80	3	60.00%	88.57%	12.63%	97.35%	2.21	0.19
FEV1% pred.	0.809 (0.609–1)	0.027	71.5	2	60.00%	91.43%	11.55%	96.00%	2.55	0.29
			87.5	1	80.00%	68,57%	8.92%	94.12%	7.00	0.44
			70	3	60.00%	91.43%	11.55%	96.00%	2.55	0.29

*p-value of the difference between the computed AUC and an AUC of 0.5

** Method used to choose the cut-off point: 1: nearest to the top left; 2: Youden; 3: arbitrary value used as the lower limit of normal

PPV—positive predictive value, NPV—negative predictive value, LR—likelihood ratio; FVC- Forced Vital Capacity, FEV1 -Forced Expiratory Volume in 1s

## Discussion

We found that CAM might be a useful alternative treatment in respiratory sufficient COP patients. Our main findings were: Patients with a FVC >80%, the predicted value treated with CAM, have a high probability of response. CAM treatment is shorter than steroid treatment, and is associated with fewer adverse events and relapses. However, CAM should be administered with caution. Notably, in our study patients with secondary organizing pneumonia due to connective tissue disease, active infectious disease or malignant disease were excluded. In addition, patients with severe impairment of pulmonary function and signs of respiratory insufficiency were excluded. The findings of our study should not be conferred upon patients not fulfilling the inclusion criteria.

### Study population

Our patient population did not differ markedly from those of previous series. COP is a rare disease, occurring in 6 to 7 people per 100 000 hospitalizations but the morbidity is underestimated because a lung biopsy is essential for an OP diagnosis [2,3]. All presented cases had histologically confirmed diagnoses (mainly via open lung biopsy). The material that we studied had been collected over many years [1–3, 9–12]. It is a disease of the fifth or sixth decade of life; our patients were mainly of this age [1–3,12–20]. In many patient series the disease affected men and women equally, but in others, as in our present work, the disease was reported more frequently in women [1–20]. We did not find that smoking was related to COP development; others have reported similar findings [2,12,17,18]. Usually, literature data on concomitant diseases have appeared in reports of patients with secondary OP [1, 13, 14, 17, 18, 25, 26]. Common conditions in such patients include arterial hypertension, atrial fibrillation, asthma, diabetes mellitus, and cardiac failure [1,17,13,25]. Gaillety et al. [20] recently reported that gastroesophageal reflux was evident in about half of all OP patients but, in our present study, only 10% of patients had this condition. This suggests that the gastrointestinal tract should be carefully examined, even when GERD symptoms are not prominent. Concomitant diseases were seen in 53% of our patients, with thyroid disease being the most common. A connection between thyroid disease and OP has been previously suggested; therefore, we paid special attention to the thyroid [37]. A detailed examination of the thyroid is not within the spectrum of the typical examination of patients with OP patients. Obscure, asymptomatic thyroid disease was diagnosed in 13% of patients, but 21% patients had clinical symptoms, mainly hypothyroidism; only 2(3%) patients developed hyperthyroidism simultaneously with OP. It is possible that thyroid disease may affect the development of OP; however, further data are required to examine this connection.

#### Clinical manifestations

Similar to other reports, we encountered principally nonspecific clinical manifestations of OP, such as a subacute flu-like syndrome [1–20]. Usually, the first symptoms preceded the diagnosis by 2 to 3 months; as in other studies [3,4,5,14,17]. The duration of symptoms in patients treated with CAM was shorter than in patients treated with PRE (3.15 vs. 3.59 months), but the difference was not significant. A shorter therapy delay and “good” clinical status might influence favorable treatment outcomes. It has been suggested that delayed treatment increases the risk of relapses; however, relapses did not affect the clinical outcomes [12].

#### Radiological manifestations

The most typical radiological abnormalities of COP are bilateral peripheral infiltrates with air bronchogram that can migrate spontaneously. Nodular, focal, or reticulonodular changes; pleural fluid; and lymph node enlargement are rarely seen [1–26]. Imaging findings, consistent with those mentioned above, were apparent in our patients [38]. However, in our present study, ground glass opacities and nodular lesions were more frequent in patients treated with CAM than PRE, but this was not associated with a higher risk of relapse or treatment failure.

#### Laboratory findings

The value of laboratory findings in diagnosing COP is generally small [1–26]. As in other inflammatory diseases, essential elevation of the erythrocyte sedimentation rate and leukocytosis were seen. In about 14% of patients, the presence of low levels of autoantibodies without symptoms of connective tissue disease was found; this was also reported in other series of patients [15,17,18].

#### Pulmonary function tests

Frequently, the ventilatory disorders of our OP patients were not severe; a decrease in the diffusing capacity of the lungs for carbon monoxide was the most common disturbance [1–18]. Lesions causing restrictive and obstructive lung disease were evident in 12 and 18% of patients, respectively; however a decrease in the diffusing capacity of the lungs for carbon monoxide was evident in 45%. This said, we emphasize that only respiratorily adequate patients were analyzed.

### Treatment and relapses

Steroids are generally accepted to be the standard treatment for COP. However, no optimal treatment modality has been precisely defined. At the beginning of our study interval, the prednisone dose was higher (1 mg/kg/d) and the treatment duration longer (> 1 year) than later. Subsequent patients received lower doses of prednisone, usually 0.5 mg/kg/d, for shorter periods (about 6 months). Lazor et al. suggested administration of 0.75 mg/ kg/d prednisone for 4 weeks, then 0.5 mg/kg/d for 4 weeks, then 20 mg/d for 4 weeks, then 10 mg/d for 6 weeks, and finally 5 mg/d for 6 weeks [12]. Epler et al. commenced with 1 mg/kg/d prednisone (maximum 60 mg/d) for 1–3 months, then switched to 40 mg/d for 3 months, and finally to 10 to 20 mg/d for a total of 1 year [8]. King et al. suggested initial therapy with 1–1.5 mg/kg/d prednisone for 4–8 weeks, then tapered to 0.5–1 mg/kg/d for 4–6 weeks, followed by a further reduction [5, 6]. Evidence is growing that other anti-inflammatory therapies may be effective in COP patients; macrolides may be valuable [21–26]. Macrolides have both specific and nonspecific anti-inflammatory effects in patients with diffuse panbronchiolitis [28], cystic fibrosis [29], asthma [30], bronchiectasis [31], and bronchiolitis obliterans developing after lung transplantation [32]. The efficacy of macrolides in OP has been demonstrated in a few studies. Ichikawa et al. [22] reported six patients with COP treated successfully with erythromycin over 3 to 4 months. Epler et al. [23] described a patient with OP treated with a macrolide. Stover and Mangino [24] reported three patients with COP and three with radiation-related OP who were treated with CAM. Ding et al. [21] recently presented a summary of 35 published cases involving patients with OP treated with macrolides, mostly CAM; among them were the ones mentioned above and our previously published data. The treatment was predominantly beneficial, but it failed in some cases. We found that patients, with FVCs >80% the predicted values, can be treated with CAM. Frequently, the response to PRE is rapid, indeed spectacular. CAM resolved both symptoms and radiological lesions less rapidly than PRE but significant improvements were usually evident after 1 month. Previous data showed that 20% to 50% of patients with OP relapsed [2,5,12,13,18,23]. A similar percentage of relapses was noted in our patients treated with PRE, but only 8% of patients treated with CAM relapsed. We also observed that relapses after failure of PRE or CAM treatment were successfully treated with CAM. A similar observation was made by Pathak et al. [26] in four patients with COP treated with steroids and subsequently with CAM. We found that all patients responded to initial PRE treatment, but CAM was ineffective in some (12%). Also, Lazor et al. [12], Costabel et al. [4], and Sveinsson et al. [16] observed no incident of failure of initial corticosteroid treatment, but 3–14.5% of patients evaluated by Cazzato et al. [11], Oymak et al. [14], Izumi et al. [3], and Yoo et al. [18] developed acute COP exacerbations and died. However, these studies were performed many years ago; some patients had secondary OP, and (probably) a few actually had acute fibrinous organizing pneumonia. Usually, the patients were of poor clinical status and many had miserable prognoses. Evaluation of possible secondary causes of organizing pneumonia is essential when selecting patients for CAM treatment. Patients with non-typical radiological changes underwent open lung biopsy. Frequently, nonspecific interstitial pneumonia overlapping with organizing pneumonia was apparent. On the other hand, patients with radiological suggestions of organizing pneumonia that were not confirmed histologically because the disease was rapidly progressive, and associated with respiratory insufficiency, were not enrolled in the present study. These various factors may explain why our treatment results were better than earlier reported.

#### Adverse events

Thirty-six percent of patients treated with corticosteroids (PRE) developed adverse events, some of which were very serious, including gastrointestinal bleeding, bone fracture, diabetes mellitus, arterial hypertension, and body weight increase. Such severe events were more evident near the time of study commencement, when patients were treated with higher doses of PRE for longer times, and protective proton-pump inhibitors and bisphosphonates were not available. Lazor et al. [12] reported that 25% of patients experienced one or more complications of corticosteroid treatment. Drakopanagiotakis et al. [17] observed a 1-year mortality rate of about 5.3% among patients with COP. An extremely high percentage of deaths was reported in patients with COP by Yoo et al. [18]. Other investigators have reported deaths during the course of COP, usually due to acute exacerbation after discontinuation of prednisone or due to adverse events, such as pulmonary embolism or pneumothorax [14,11]. In contrast, there was a single allergic reaction in one patient treated with CAM. It has been suggested that macrolides may increase the number of cardiac events. However, the extensive analysis of Chou et al. found no association between clarithromycin use and adverse cardiac outcomes [42]. Our CAM-treated group was rather small. Only a few patients were aged >70 years or had coronary heart disease; no patient had a circulatory insufficiency or cardiac arrhythmia. Thus, the relatively good condition of our patients may explain the lack of cardiac events.

This was an observational, retrospective, single-center work. The study material had been collected over many years, and we selected only patients of good clinical status who were respiratorily adequate. Patients were treated with different doses of PRE and with different therapy durations, but CAM was administered using a standardized regimen. Thus, a multicenter, randomized study is necessary to establish the role of CAM in OP treatment.

## Conclusions

CAM may be a useful alternative for patients with COP and radiotherapy-induced OP who have normal FVC and FEV1 values. The course of CAM therapy was shorter than that of corticosteroid therapy, and was associated with fewer relapses and adverse events, but CAM was ineffective in some patients. A future prospective randomized study is essential.

## Supporting information

S1 Fig ROC for clarithromycin treatment failure vs FVC as %pred. (TIFF)

S2 Fig ROC for clarithromycin treatment failure vs FEV1 as %pred. (TIFF)

S1 File (DOCX)

S2 File (XLS)
