# Supplementary material for: Cryptogenic organizing pneumonia—Results of treatment with clarithromycin versus corticosteroids—Observational study
Source: PLoS One. 2017 Sep 25;12(9):e0184739. doi: 10.1371/journal.pone.0184739 (PMC5612459; doi:10.1371/journal.pone.0184739)
Supplement: S1 File — (DOCX) [file pone.0184739.s003.docx]

Supplement

Table 1a.

Single variant regression analysis factors influence on treatment failure and/or relapse in the group of all patients

| **Variable** | **OR** | **-95%CI** | **+95%CI** | ***p*** |
| --- | --- | --- | --- | --- |
| Sex | 0.963 | 0.281 | 3.292 | 0.951 |
| Smoking | 0.243 | 0.028 | 2.097 | 0.198 |
| Age | 0.991 | 0.940 | 1.046 | 0.754 |
| Weight loss | 0.632 | 0.213 | 1.881 | 0.410 |
| Dyspnea | 2.270 | 0.728 | 7.078 | 0.158 |
| Weakness | 2.368 | 0.257 | 21.787 | 0.446 |
| Cough | 1.616 | 0.390 | 6.698 | 0.508 |
| Sputum | 0.923 | 0.273 | 3.123 | 0.897 |
| Chest pain | 0.824 | 0.246 | 2.766 | 0.754 |
| Sweat | 0.671 | 0.221 | 2.032 | 0.480 |
| Fever | 0.574 | 0.170 | 1.932 | 0.370 |
| Hypertension | 0.779 | 0.247 | 2.455 | 0.670 |
| Depression | 2.333 | 0.138 | 39.394 | 0.557 |
| GERD | 0.343 | 0.038 | 3.063 | 0.338 |
| Brest cancer | 0.741 | 0.072 | 7.618 | 0.801 |
| Ischemic heart disease | 0.741 | 0.072 | 7.618 | 0.801 |
| Asthma | 4.941 | 0.420 | 58.168 | 0.204 |
| COPD | 0.000 | 0.000 | Inf | 0.991 |
| Diabetes | 7.875 | 0.762 | 81.370 | 0.083 |
| Goiter | 1.506 | 0.496 | 4.580 | 0.470 |
| Migration of lesions | 0.918 | 0.299 | 2.825 | 0.882 |
| Infiltrations | 2604724.855 | 0.000 | Inf | 0.992 |
| Nodules | 0.370 | 0.105 | 1.304 | 0.122 |
| Reticular lesions | 1.644 | 0.405 | 6.670 | 0.486 |
| Lymph nodes enlargement | 2.600 | 0.575 | 11.751 | 0.214 |
| Pleural fluid | 0.000 | 0.000 | Inf | 0.993 |
| Ground glass opacities | 1.616 | 0.390 | 6.698 | 0.508 |
| ERS | 1.008 | 0.992 | 1.024 | 0.340 |
| CRP | 1.002 | 0.989 | 1.015 | 0.759 |
| WBC | 0.884 | 0.699 | 1.119 | 0.305 |
| EOS | 0.025 | 0.000 | 2.938 | 0.129 |
| **FVC% pred.** | **0.934** | **0.898** | **0.972** | **0.001** |
| TLC% pred. | 0.998 | 0.965 | 1.033 | 0.922 |
| FEV1% pred. | 0.989 | 0.958 | 1.021 | 0.492 |
| FEV1%VC | 1.023 | 0.977 | 1.070 | 0.332 |
| RV% pred. | 0.996 | 0.965 | 1.027 | 0.795 |
| DLCO% pred. | 1.004 | 0.970 | 1.040 | 0.810 |
| PaO2 mmHg | 0.965 | 0.903 | 1.031 | 0.291 |
| PaCO2 mmHg | 0.893 | 0.749 | 1.065 | 0.207 |
| RF | 1.139 | 0.097 | 13.379 | 0.918 |
| ANA | 1.543 | 0.325 | 7.334 | 0.586 |
| Antithyroid peroxidase antibodies | 4.154 | 0.672 | 25.683 | 0.126 |
| Antithyreoglobulin antibodies | 3.733 | 0.312 | 44.628 | 0.298 |
| **TBLB** | **0.102** | **0.021** | **0.498** | **0.005** |
| **Adverse events** | **30.545** | **3.445** | **270.828** | **0.002** |
| Hypertension | 121961795.380 | 0.000 | Inf | 0.992 |
| **Increase of body weight** | **11.200** | **1.158** | **108.330** | **0.037** |
| Diabetes | 39588736.117 | 0.000 | Inf | 0.992 |
| **Upper respiratory tract infection** | **24.500** | **2.738** | **219.206** | **0.004** |
| Pulmonary embolism | 13754775.165 | 0.000 | Inf | 0.991 |
| Urinary tract infection | 2.333 | 0.138 | 39.394 | 0.557 |
| Bone fracture | 13754775.167 | 0.000 | Inf | 0.991 |
| Time to diagnosis | 0.819 | 0.612 | 1.096 | 0.179 |
| **Observation time** | **1.025** | **1.006** | **1.045** | **0.010** |
| Treatment failure | 130673352.185 | 0.000 | Inf | 0.992 |
| **Relapse** | 3186306660.925 | 0.000 | Inf | 0.994 |

ANA Antinuclear antibody

COPD Chronic obstructive pulmonary disease

CRP C reactive protein serum concentration

EOS Eosinophil count

ESR Erythrocyte sedimentation rate

GERD Gastro-esophageal reflux disease

RF Rheumatoid factor

TBLB Transbronchial lung biopsy

WBC White blood cells count

FVC Forced Vital Capacity

FEV1 Forced Expiratory Volume in 1 s
TLC Total Lung Capacity

RV Residual Volume

DLCO Diffusion Lung Capacity for Carbon Monoxide

PaO2 Partial pressure of oxygen in arterialized blood

PaCO2 Partial pressure of carbon dioxide in arterialized blood

CI Confidence interval

OR Odds ratio

Table 2a.

Single variant regression analysis factors influence on treatment failure and/or relapse in the group of patients treated with CLA

| **Variable** | **OR** | **-95%CI** | **+95%CI** | ***p*** |
| --- | --- | --- | --- | --- |
| Sex | 0.673 | 0.107 | 4.238 | 0.673 |
| Age | 0.990 | 0.911 | 1.077 | 0.819 |
| Smoking | 0.000 | 0.000 | Inf | 0.995 |
| Weight loss | 0.553 | 0.106 | 2.873 | 0.481 |
| Dyspnea | 1.111 | 0.214 | 5.764 | 0.900 |
| Weakness | 9606893.157 | 0.000 | Inf | 0.995 |
| Cough | 1.615 | 0.166 | 15.724 | 0.680 |
| Sputum | 0.920 | 0.152 | 5.566 | 0.928 |
| Chest pain | 0.383 | 0.041 | 3.612 | 0.402 |
| Sweat | 1.111 | 0.214 | 5.764 | 0.900 |
| Fever | 0.427 | 0.078 | 2.325 | 0.325 |
| Hypertension | 1.018 | 0.196 | 5.292 | 0.983 |
| Depression | 5.333 | 0.292 | 97.490 | 0.259 |
| GERD | 0.000 | 0.000 | Inf | 0.995 |
| Breast cancer | 0.000 | 0.000 | Inf | 0.994 |
| Ischemic heart disease | 0.000 | 0.000 | Inf | 0.994 |
| Asthma | 5.333 | 0.292 | 97.490 | 0.259 |
| COPD | 0.000 | 0.000 | Inf | 0.995 |
| Diabetes | 0.000 | 0.000 | Inf | 0.995 |
| Goiter | 2.051 | 0.393 | 10.700 | 0.394 |
| Migration of lesions | 0.937 | 0.153 | 5.728 | 0.944 |
| Nodules | 0.425 | 0.072 | 2.511 | 0.345 |
| Reticular lesions | 2.240 | 0.336 | 14.916 | 0.404 |
| Lymph nodes enlargement | 2.583 | 0.201 | 33.242 | 0.467 |
| Pleural fluid | 0.000 | 0.000 | Inf | 0.995 |
| Ground glass opacities | 9606893.050 | 0.000 | Inf | 0.995 |
| RF | 0.000 | 0.000 | Inf | 0.995 |
| ANA | 0.933 | 0.092 | 9.508 | 0.954 |
| Antithyroid peroxidase antibodies | 4.400 | 0.494 | 39.211 | 0.184 |
| Antithreoglobulin antibodies | 3.833 | 0.208 | 70.639 | 0.366 |
| TBLB | 0.200 | 0.033 | 1.201 | 0.078 |
| Time to diagnosis | 0.812 | 0.489 | 1.348 | 0.421 |
| Observation time | 1.021 | 0.987 | 1.055 | 0.224 |

ANA Antinuclear antibody

COPD Chronic obstructive pulmonary disease

GERD Gastro-esophageal reflux disease

RF Rheumatoid factor

TBLB Transbronchial lung biopsy

CI Confidence interval

OR Odds ratio

Table 3a.

Single variant regression analysis factors influence relapse in the group of patients treated with PRE

| **Variable** | **OR** | **-95%CI** | **+95%CI** | ***p*** |
| --- | --- | --- | --- | --- |
| Sex | 2.000 | 0.324 | 12.329 | 0.455 |
| Age | 1.018 | 0.938 | 1.105 | 0.661 |
| Smoking | 0.212 | 0.018 | 2.467 | 0.215 |
| Weight loss | 1.071 | 0.194 | 5.913 | 0.937 |
| **Dyspnoe** | **7.000** | **1.067** | **45.903** | **0.043** |
| Weakness | 4.714 | 0.405 | 54.828 | 0.215 |
| Cough | 2.143 | 0.281 | 16.370 | 0.463 |
| Sputum | 1.333 | 0.176 | 10.121 | 0.781 |
| Pain | 1.167 | 0.191 | 7.117 | 0.867 |
| Sweat | 1.333 | 0.176 | 10.121 | 0.781 |
| Fever | 0.333 | 0.029 | 3.842 | 0.378 |
| Hypertension | 1.333 | 0.176 | 10.121 | 0.781 |
| GERD | 0.818 | 0.045 | 14.996 | 0.892 |
| Breast Cancer | 14228509.809 | 0.000 | Inf | 0.995 |
| Ischemic heart disease | 14228509.809 | 0.000 | Inf | 0.995 |
| COPD | 0.000 | 0.000 | Inf | 0.994 |
| Diabetes | 47272013.712 | 0.000 | Inf | 0.994 |
| Goiter | 4.500 | 0.413 | 49.079 | 0.217 |
| Migration of lesions | 2.100 | 0.381 | 11.589 | 0.395 |
| Nodules | 0.800 | 0.091 | 7.002 | 0.840 |
| Reticular lesions | 1.800 | 0.139 | 23.375 | 0.653 |
| Lymph nodes enlargement | 1.333 | 0.176 | 10.121 | 0.781 |
| **Ground glass opacities** | **12.000** | **1.581** | **91.087** | **0.016** |
| ERS | 1.011 | 0.987 | 18.201 | 0.370 |
| CRP | 0.983 | 0.954 | 0.988 | 0.238 |
| WBC | 0.753 | 0.495 | 1.018 | 0.186 |
| EOS | 0.004 | 0.000 | 1.006 | 0.211 |
| **FVC%pred.** | **0.935** | **0.885** | 1.088 | **0.018** |
| TLC %pred. | 1.042 | 0.976 | 1.025 | 0.219 |
| FEV1% pred. | 1.019 | 0.975 | 1.037 | 0.407 |
| FEV1%VC | 1.013 | 0.940 | 1.011 | 0.741 |
| RV% pred. | 1.031 | 0.958 | 1.210 | 0.416 |
| DLCO% pred. | 1.018 | 0.968 | 1.348 | 0.483 |
| PaO2 mmHg | 0.997 | 0.919 |  | 0.934 |
| PaCO2 mmHg | 0.891 | 0.690 |  | 0.376 |
| RF | 0.818 | 0.045 | 14.996 |  |
| Antithyroid peroxidase antibodies | 26590507.724 | 0.000 | Inf |  |
| Antithyreoglobulin antibodies | 23636006.855 | 0.000 | Inf |  |
| TBLB | 0.000 | 0.000 | Inf |  |
| **Adverse events** | **18.000** | **1.650** | **196.309** | **0.018** |
| Hypertension | 144560990.914 | 0.000 | Inf | 0.995 |
| Increase of body weight | 4.500 | 0.413 | 49.079 | 0.217 |
| Diabetes | 42544812.339 | 0.000 | Inf | 0.995 |
| **Upper respiratory tract infection** | **12.600** | **1.186** | **133.897** | **0.036** |
| Urinary tract infections | 0.818 | 0.045 | 14.996 | 0.892 |
| Bone fracture | 14228509.809 | 0.000 | Inf | 0.995 |
| Time to diagnosis | 0.722 | 0.482 |  | 0.115 |
| **Observation time** | **1.031** | **0.997** | **1.065** | **0.072** |

ANA Antinuclear antibody

COPD Chronic obstructive pulmonary disease

CRP C reactive protein

EOS Eosinophil count

ERS Erythrocyte sedimentation rate

GERD Gastro-esophageal reflux disease

RF Rheumatoid factor

TBLB Transbronchial lung biopsy

WBC White blood cells count

FVC Forced Vital Capacity

FEV1 Forced Expiratory Volume in 1 s
TLC Total Lung Capacity

RV Residual Volume

DLCO Diffusion Lung Capacity for Carbon Monoxide

PaO2 Partial pressure of oxygen in arterialized blood

PaCO2 Partial pressure of carbon dioxide in arterialized blood

CI confidence interval

OR odds ratio

For the assessment of relative goodness of fit for one- and bifactorial logit models, an Akaike information criterion (AIC) was used. The smaller AIC predicts the better model.

Table 4s.

Probability equations and Akaike information criterion (AIC) value for single- and bifactorial logit models.

| Factors | Logit model, probability of treatment failure | AIC |
| --- | --- | --- |
| FVC% pred. | P=1/(1+exp(0.10015*FVC-6.4224)) | 21.548 |
| FEV1% pred | P=1/(1+exp(0.08351*FEV1-4.98177)) | 28.155 |
| FVC% pred, FEV1% pred | P=1/(1+exp(0.09295*FVC+0.04555*FEV1-9.52094)) | 22.591 |

FVC Forced Vital Capacity

FEV1 Forced Expiratory Volume in 1 s

The difference between uni and bivariate models with FVC% pred. was small but still univariate model was better fitted. It confirms that FVC% pred. was the main factor determining response to CAM treatment in our group.

The difference between uni- and bivariate models with FVC% pred. was very small but still univariate model was better fitted. It confirms, that FVC% pred. was the main factor determining response to CAM treatment in our group.

**Fig 1. Receiver Operating Curve (ROC) for FVC% pred. in patients treated with clarithromycin**

AUC area under the curve

FVC Forced Vital Capacity

**Fig 2. Receiver Operating Curves (ROC) for FEV1%pred. in patients treated with clarithromycin**

AUC area under the curve

FEV1 Forced Expiratory Volume in 1 s

|  |  |  |  | **0.040** |
| --- | --- | --- | --- | --- |
|  |  |  |  | **0.083** |
|  |  |  |  | **0.084** |
